# Trans-National Scale-Up of Services in Global Health

**DOI:** 10.1371/journal.pone.0110465

**Published:** 2014-11-06

**Authors:** Ilan Shahin, Raman Sohal, John Ginther, Leigh Hayden, John A. MacDonald, Kathryn Mossman, Himanshu Parikh, Anita McGahan, Will Mitchell, Onil Bhattacharyya

**Affiliations:** 1 Women's College Hospital, University of Toronto, Toronto, ON, Canada; 2 Institute of Health Policy Management and Evaluation, University of Toronto, Toronto, ON, Canada; 3 Toronto Health Organization Performance Evaluation (T-HOPE), University of Toronto, Toronto, ON, Canada; 4 MIT Media Lab and Sloan School of Management, Boston, MA, United States of America; 5 Rotman School of Management, University of Toronto, Toronto, ON, Canada; 6 Department of Family Medicine, University of Toronto, Toronto, ON, Canada; Stanford University, United States of America

## Abstract

**Background:**

Scaling up innovative healthcare programs offers a means to improve access, quality, and health equity across multiple health areas. Despite large numbers of promising projects, little is known about successful efforts to scale up. This study examines trans-national scale, whereby a program operates in two or more countries. Trans-national scale is a distinct measure that reflects opportunities to replicate healthcare programs in multiple countries, thereby providing services to broader populations.

**Methods:**

Based on the Center for Health Market Innovations (CHMI) database of nearly 1200 health programs, the study contrasts 116 programs that have achieved trans-national scale with 1,068 single-country programs. Data was collected on the programs' health focus, service activity, legal status, and funding sources, as well as the programs' locations (rural v. urban emphasis), and founding year; differences are reported with statistical significance.

**Findings:**

This analysis examines 116 programs that have achieved trans-national scale (TNS) across multiple disease areas and activity types. Compared to 1,068 single-country programs, we find that trans-nationally scaled programs are more donor-reliant; more likely to focus on targeted health needs such as HIV/AIDS, TB, malaria, or family planning rather than provide more comprehensive general care; and more likely to engage in activities that support healthcare services rather than provide direct clinical care.

**Conclusion:**

This work, based on a large data set of health programs, reports on trans-national scale with comparison to single-country programs. The work is a step towards understanding when programs are able to replicate their services as they attempt to expand health services for the poor across countries and health areas. A subset of these programs should be the subject of case studies to understand factors that affect the scaling process, particularly seeking to identify mechanisms that lead to improved health outcomes.

## Introduction

Many effective and inexpensive health interventions could address the burden of disease in low and middle-income countries (LMICs), but population coverage is poor due to major gaps in delivery [1], [2]. Health systems in many LMICs include a large private sector, comprised of a mix of licensed for-profit and nonprofit organizations, as well as informal providers [3]. Some of these private-sector organizations have developed viable approaches to provide affordable, accessible, and quality healthcare [4], [5]. Understanding the potential for replication and scale up of these approaches is important, but currently remains unclear, both within health systems and across different countries [6].

Understanding scaling up is critical to extend the reach of health services programs with clinically effective models that are cost-efficient and financially sustainable for people who have limited purchasing power, live in underserved areas, and have low health literacy [4], [7]. The medical and economic value of health services programs that have scaled can also make them attractive to governments, donors, and investors in search of solutions to address urgent global health problems. This paper describes more than one hundred healthcare programs that operate in multiple countries, seeking to identify common characteristics of such programs. The study focuses on one dimension of scale, geographic coverage, which is the ability of a program to replicate its model in another country. The study provides a starting point for investigating other aspects of scale as well as when scaled up programs are able to provide high quality healthcare services.

Subramanian *et al*. [8] observe that the predominant focus in the global health field is on achieving high coverage rates of health services, and reducing mortality to the neglect of understanding the processes for how to scale up. Scaling healthcare services involves multiple potential dimensions. The Scaling Up Management (SUM) Framework from Management Systems International, perhaps the most general framework for assessing scale, suggests that a program can scale its services in several ways, including: *breadth of coverage* (expanding to cover more people in the currently served area); *depth of services* (offering additional services to current clients); *client type* (expanding services to new categories of clients); *problem definition* (expanding current methods to new problems and health areas); and *geographic coverage* (expanding to new locations) [9]. Research has only begun to examine the nature and determinants of these forms of scaling. While some literature addresses scaling in the public and private healthcare sectors, much of this work assesses specific disease areas and it is unclear how to generalize the findings [10].

This paper examines expansion of geographic coverage in the form of trans-national scale, which we define as health programs that operate in more than one country. Trans-national scale indicates broader replicability compared to programs that operate within one country, demonstrating that a program and its innovations can be implemented in heterogeneous settings.

Our goal in this paper is to help identify factors that contribute to scaling up, both to determine current patterns and to identify potential routes for new opportunities for scaling. The achievement of trans-national scale does not necessarily match to health quality and/or outcomes; and issues related to performance and impact need to be the focus of additional research. Nonetheless, multi-country replication warrants examination, because the ability of a program to transplant its model from one context to another context, which typically includes different socio-economic, cultural, and political aspects, signals relevant aspects of the replicability of the model and its ability to address health challenges on a larger scale as compared to single-country initiatives.

The study draws on the Center for Health Market Innovations database of nearly 1,200 private health services providers in LMICs. CHMI defines innovations as “programs and policies – implemented by governments, non-governmental organizations (NGOs), social entrepreneurs, or private companies – that improve privately delivered healthcare”, including organizing delivery, financing care, regulating performance, changing behaviors, and enhancing processes (CHMI website). We examine programs in the CHMI database that have achieved trans-national scale (TNS) and compare them to single country programs (SCPs). In cataloguing cases of trans-national scale, our research identifies key characteristics of programs that are able to operate in multiple countries. These findings illuminate factors that have facilitated or constrained trans-national scale and offer insights for scholars, policy-makers, funders, investors, and program managers seeking to identify scalable solutions capable of providing broader health impact.

## Methods

The Center for Health Market Innovations is managed by the Results for Development Institute, which curates a database of organizations dedicated to improving privately delivered healthcare for the poor in LMICs. Sixteen regional partners recruit organizations in LMICs to submit data. The data include information about programs offered by organizations in LMICs that attempt to improve access, quality, and/or affordability of health services through activities such as direct patient contact, financial interventions, and supply chain support. Although providing cross-sectional rather than longitudinal information, the CHMI data provides the most general available set of comparative data about private-sector healthcare organizations operating in LMICs. This study uses programs as the unit of analysis, where a program is an operating entity that functions with a particular scope of objectives. For most cases in the data, each parent organization (in some cases, a partnership of multiple organizations) operates a single program.

This study examines selected programs in the CHMI database that reported a presence in two or more countries, and contrasts their characteristics with programs that operate within only one country. We extracted information from the database based on four main program characteristics: health focus, activity, legal status, and funding source. *Health focus* refers to the health needs a program targets (e.g., family planning and reproductive health, HIV/AIDS); *Activity* refers to the program's service offerings wherein lies its innovation (e.g., provider training, information technology, consumer outreach); *Legal status* refers to how a program's parent organization is registered for ownership status (e.g., private for-profit, private not-for-profit, government); *Funding source* refers to the sources of capital for the program (e.g., donor, government, revenue-generation). Within the CHMI reporting framework, programs may provide one or more responses for health focus and funding source; they report a single category for activity and legal status. The database has extensive coverage of these four characteristics; the rate of reported results for health focus, activity, legal status, and funding sources ranged from 88% to 100%. We assessed differences for the four characteristics between TNS and single country programs based on descriptive statistics and t-tests of subsample means. We examined the characteristics of outlier programs that operated in ten or more countries. We also examined whether TNS status is associated with country and regional location, rural v. urban coverage, or founding year.

Thus, in examining TNS programs, the study focuses on replication of a healthcare model beyond a single country. We stress that the study provides insight into *what* TNS programs are doing, without being able to asses *why* and *how* TNS programs scale. Nonetheless, understanding the characteristics of healthcare programs that achieve TNS scale is of interest to program managers who seek to expand their activities, as well as to donors and investors who seek to identify and invest in programs able to reach as many people as possible, working across a range of resource constrained-settings [4]. Further research with additional data can examine other important aspects of scale, such as program scope and quality of service.

## Results

Screening for programs in the CHMI database that operate in two or more countries identified 116 distinct programs operating in 90 unique countries; trans-national programs operated in a median of 3 countries, with a range of 2 to 22 countries. The African continent (Sub-Saharan and North Africa) had the largest share, with 95 programs (i.e., 82% of the 116 TNS programs operated in at least one African country), while Asia had 49 (42% of the TNS programs) and the Americas (Latin America and the Caribbean) had 25 (22%) programs. The majority (65%) of the TNS programs operated in a single continent, but 25% operated in two continents and another 10% operated across the three continental areas. The 116 TNS programs were founded as early as 1952 (the Sightsavers program, which provides eye care in Kenya, Sudan, Tanzania, and Uganda) and as late as 2012, with a median founding year of 2006.

**Figure 1:**
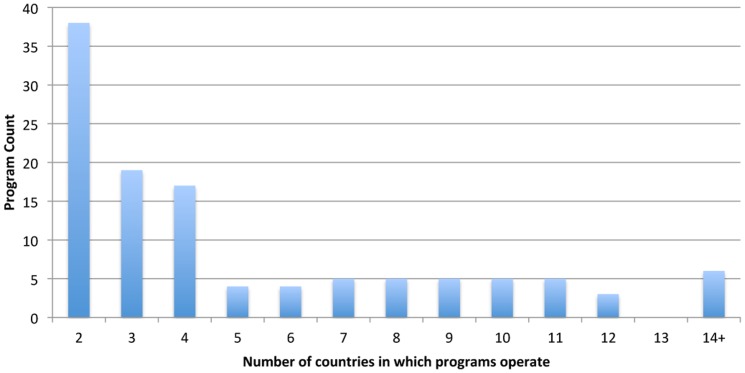
Program count for number of countries of operation.

Figure 1 shows the distribution of TNS programs by TNS breadth, i.e., the number of countries they operate in: 38 (33%) operate in two countries, another 36 (31%) operate in three or four countries, 28 (24%) operate in five to ten countries, and the remaining 14 (12%) operate in more than ten countries. Thus, most TNS programs are limited to a few countries but a meaningful number achieve substantial international breadth, across countries and continents.

### Health focus

**Figure 2:**
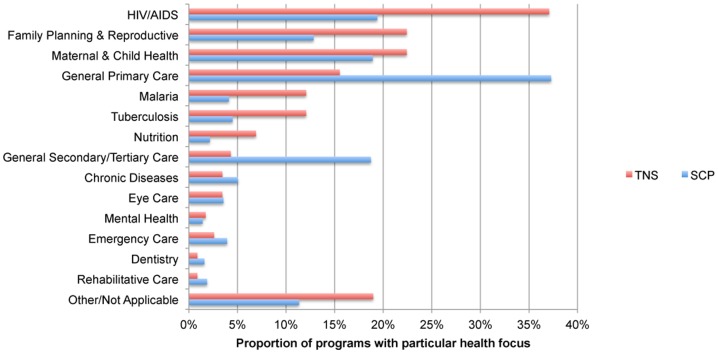
Distribution of health focus of trans-national scale (TNS) and single-country programs (SCP).

Figure 2 compares the health focus of the 116 TNS programs with the 1,068 single country programs (SCP) we identified in the database. The key implication of the comparison is that TNS programs are particularly likely to target specific health needs, whereas SCPs are more likely to provide general care.

The figure shows that more than a third of the TNS programs offer HIV/AIDS services (37%), followed by family planning & reproductive health (22%) and maternal and child health (22%). This contrasts with SCPs, where general primary care leads with 37% of responses (versus 16% in TNS), while HIV/AIDS represents the focus for only 19% of single country programs.

The data demonstrate that more than half of the TNS programs target specific health needs such as malaria, TB, HIV/AIDs, and family planning. For instance, D-Tree International, which was founded in 2004, has expanded from Tanzania to Malawi, South Africa, and India, providing clinical protocols via decision support software on mobile phones for use by clinic staff and community health workers to help them assess, diagnose and treat patients. The protocols address a substantial range of targeted services (e.g. HIV/AIDS, family planning, maternal and child health [MCH], TB, malaria, and chronic diseases).

We also examined whether programs offered single or multiple services. Single service programs are most common: 65% of TNS programs report a single focus out of 15 possible categories in the survey (mean of 1.65 health focus responses) and 66% of SCPs report a single focus (mean of 1.47 health focus responses). Among multi-service programs, TNS programs are most likely to offer combinations of HIV/AIDS, MCH, family planning and reproductive health, TB, and/or malaria services (73% of the 41 multi-service TNS v. 58% of the 366 multi-service SCPs), whereas multi-service SCPs are most likely to offer combinations that include general primary care and/or secondary/tertiary care (66% of the multi-service SCPs v. 10% of the multi-service TNS programs). This comparison reinforces the distinction in which TNS programs address targeted needs, while SCPs address more general clinical care.

### Activities

Table 1 shows the types of support activities SCP and TNS programs have adopted for their health services. Among TNS programs, the most common activities included information technology (35%) and consumer outreach (34%), as well as multiple forms of delivery support (48%), including provider training, operational processes, equipment, and supply chain enhancement. SCPs provide similar levels of IT (27%) and consumer outreach (28%), but significantly lower levels of delivery support (25%). TNS programs also were more likely to provide diagnostic testing (8% v. 2%) and to expand via franchising (9% v. 4%).

**Table 1:** Frequency of SCP and TNS program activities.

Activities	SCP	TNS
A. Information technology (IT)	27%	35%
B. Consumer outreach (education, social marketing)	28%	34%
C. Delivery support (at least one sub-category)	25%	48% **
C1. Provider training	17%	28% *
C2. Innovative operational processes	6%	15% **
C3. Products/equipment	4%	14% **
C4. Supply chain enhancements	2%	11% **
D. Diagnostics/lab testing	2%	8% *
E. Franchise	4%	9% *
F. Clinics/hospitals (mobile &/or standalone)	18% **	7%
F1. Standalone clinic/hospital	10% **	0%
F2. Mobile clinics	9%	7%
G. Health insurance (community, public, or private)	16% **	3%

Cases: SCP  = 1,068; TNS  = 116.

** p<0.01; * p<0.05 (difference of mean t-tests, different populations and variances).

**Note**: The data include 30 types of program activities; Table 1 reports categories that achieve at least 8% for SCP and/or TNS programs (80% of all SCP activities; 74% of TNS activities).

**Acronyms**: MCH  =  Maternal & Child Health; FPRH  =  Family Planning & Reproductive Health; TB-Malaria combines Tuberculosis and Malaria services; PC  =  Primary Care.

By contrast, SCPs were more likely to provide clinical care through standalone clinics or hospitals (10% v. none of the TNS programs) and/or health insurance (16% v. only 3% of TNS programs). The key point here is that TNS programs tend to emphasize support services, while SCPs are more likely than TNS programs to provide clinical care.

Several examples illustrate IT, consumer outreach, and provider training activities. 100% Jeune, for instance, is a not-for-profit program founded in 2000 that uses media and interpersonal communications to promote reduction of risk-taking behavior among youth, initially in Cameroon and now also in Chad, and the Central African Republic. Additionaly, the Helping Babies Breathe non-profit program of the American Academy of Pediatrics, launched in 2010, teaches neonatal resuscitation techniques to MCH providers in India, Bangladesh, Pakistan, Kenya, and Tanzania.

### Legal status

Table 2 reports legal status. The summary point is that TNS programs are more likely to be private non-profits (72% v. 51% of SCPs) and less likely than SCPs to be public entities (3% v. 10%). We also checked the correlations of for-profit, non-profit, and public- private partnership legal status with TNS breadth (the number of countries in which programs operated), finding no meaningful relationship.

**Table 2:** Legal status of SCP and TNS programs.

Legal status	SCP	TNS
Private (not-for-profit)	51%	72% **
Private (for profit)	14%	10%
Private (unspecified)	3%	4%
Public-private partnership	21%	19%
Public: State/government	10% **	3%
Corporate program	1%	1%

Cases: SCP  = 995 (93% reporting); TNS  = 106 (91% reporting).

** p<0.01 (difference of mean t-tests, different populations and variances).

Several examples of non-profit programs are intriguing. The non-profit AAD Telemedicine Project, launched in 2010 by the American Academy of Dermatology, for instance, electronically connects primary care physicians with dermatologists in Kenya, Botswana, Egypt, and Ghana to assist with diagnoses. Another interesting not-for-profit program is Total Health Village, founded in 2008, which trains community-based health promoters to help facilitate self-empowerment within communities in eight countries within Latin America, Africa, and Southeast Asia.

Although for-profit TNS ventures are less common, several examples are noteworthy. Sproxil, for instance, enables consumers to text a barcode number on a drug's packaging and receive a response indicating whether it is genuine or counterfeit; by early 2012, this service, free to consumers, had been used over 1 million times in Nigeria and India [11]. Other examples include Project Shakti, which distributes Unilever healthcare hygiene products in Bangladesh, India, and Sri Lanka, as well as the SAHEL venture that offers a satellite-based e-health telemedicine network to healthcare professionals and hospitals in Kenya and Senegal.

Although unusual, the few public TNS programs are also interesting. The West Africa Drug Regulatory Authority Network (WADRAN), for instance, is a multi-national collaboration that has had notable success in removing counterfeit and substandard drugs from the market. The Tanzania-Ghana Health Partnership is another collaboration between the health service ministries in these two countries that facilitates exchange and implementation of health systems strengthening and delivery models.

Although public entities rarely take on trans-national scale on their own, there is a substantial set of public-private partnerships among the TNS programs (19% similar to 21% for SCPs). One example is the Affordable Medicines Facility, which distributes drugs for Malaria in several countries in Africa. Another case is the East Africa Public Health Laboratory Networking Project, set up by public health agencies in Kenya, Uganda, Tanzania, and Rwanda to establish a network of public health laboratories to serve as surveillance sites to monitor disease transmission.

### Funding source

Table 3 reports funding sources. The key implication is that donor funding is the primary means of support for the majority of all programs, while being particularly important for programs that achieve trans-national scale.

**Table 3:** Funding source of SCP and TNS programs.

	SCP	TNS	SCP	TNS
Funding source	Any	Any	Primary	Primary #
Donor	68%	90% **	56%	82% **
Government	31% *	22%	16% **	6%
Individual: Out-of-pocket payments	24% **	14%	13%	9%
Individual: Membership/subscription fees	15% **	5%	11% **	0%
In-kind contributions	9%	9%	1%	1%
Revenue (e.g., interest on loans)	4%	6%	1%	4%
Other 3rd party (e.g., debt, equity)	6%	6%	3% *	1%

Cases: SCP  = 939 (88% reporting); TNS  = 105 (91% reporting).

** p<0.01, * p<0.05 (difference of mean t-tests, different populations and variances).

# Note: “Primary” is largest source of funding; “Any” is one of potential multiple funding sources (61% of TNS programs report having only one source of funding; compared to 33% of SCPs).

Funding among TNS programs is mostly donor-led, with 82% reporting donors as their primary funding source and 90% receiving at least some donor funding (i.e., only 10% received no donor funding). In contrast, 32% of SCPs operate independently of donors. Government funding, out-of-pocket payments, and membership fees are less common for TNS programs than for SCPs.

### Other possible relationships with trans-national scale

We investigated possible differences among the “outlier” programs that achieve substantial trans-national scale, focusing on those that operated in more than ten countries. Six programs operated in 14 or more countries: Zain Corporate AIDS Program (22 countries); Supply Change Management Systems (16); DKT International (15); Strengthening Laboratory Management Accreditation (15); AIDS Empowerment and Treatment International (14); and Venture Strategies Innovations (14). Zain is a global telecommunications company based in Kuwait that provides employees and their dependents with comprehensive HIV/AIDS counseling and care. Supply Change Management Systems (SCMS), administered by the USAID, ensures reliable, cost effective and secure supply of products for HIV/AIDS programs in developing countries. DKT International is a non-profit organization based in Washington, DC, that serves as one of the largest private providers of family planning and reproductive health products and services in the developing world. Strengthening Laboratory Management Accreditation (SLMTA), operated by the Centers for Disease Control (CDC), offers a training approach in laboratory management and quality management systems with the goal to produce measurable improvement and prepare laboratories for accreditation based on international clinical laboratory standards. AIDS Empowerment and Treatment International (AIDSETI) is a non-profit network of community-based associations founded and managed by people living with HIV/AIDS in Africa and the Caribbean; the program is affiliated with US Doctors for Africa, which provides volunteer medical personnel who educate regional staff, while providing care for individuals in the individual country associations. Venture Strategies Innovations (VSI) is a non-profit organization based in California that works with ministries of health, professional medical associations, and in-country experts to achieve regulatory approval of products that will improve women's health to integrate the products into the health system.

A few patterns stand out in the outlier analysis. Zain is a multinational corporation, which facilitates replication across countries through existing infrastructure, while SLMTA and SCMS have the support of major governmental and quasi-governmental organizations. Among the other three programs in 14 or more countries, the primary common point is they typically provide only limited clinical services, which are more difficult to scale across countries than operational or logistical activities. Five of the six programs emphasize support for targeted health needs, most commonly HIV/AIDs.

Another eight programs operate in more than ten countries. Similar to the six largest outlier TNS programs, all eight focused on targeted health needs areas (e.g., HIV/AIDs, TB, Malaria, MCH, dentistry, eye care). The focus on specific health needs by programs with greater TNS breadth might be due to donor priorities, which we address in the discussion section.

We also calculated correlations of number of countries, numbers of continents, and specific continents (Africa, Asia-Middle East, and the Americas) with both health foci and health activities, finding no meaningful patterns. The implication here is that programs that manage to extend to multiple countries can do so with a wide range of health services and activities.

We investigated two other factors that might have affected the extent of TNS breadth (number of countries): urban-rural coverage and founding year. We examined rural-urban coverage, finding that 94% of TNS programs covered rural communities, 86% covered urban communities, and 80% covered both urban and rural; hence, there was very little geographic specialization.

By contrast, founding year had a moderate relationship with TNS breadth. There was a limited positive relationship between earlier founding year and number of countries, though far from a dominant relationship. In addition, there was a moderate positive correlation between later founding year and provision of general clinical care (primary or secondary) by TNS programs (r = 0.20), perhaps suggesting a more recent emphasis on TNS general care.

## Discussion

This study analyzed a database of 1,184 low-and-middle-income country health programs and identified 116 programs that have scaled across more than one country, offering more than 20 different types of activities in 14 health service areas in 90 different countries. These 116 TNS programs were compared to 1,068 single country health programs. The study focuses on scale in terms of programs replicated by a single entity, as opposed to programs being replicated by different parent organizations. Program replication in different countries helps spread key health interventions – it is notable that almost 10% of a large sample of programs covering a range of health areas was able to achieve trans-national scale [11].

The study suggests strategies and barriers to scaling up. Much of the literature offers conceptual frameworks [12] or cases focused on specific disease areas such as malaria, mental health, and neonatal care [13], [14], [15]. Even the most systematic overview of scaling focuses on one aspect, the costs of scaling health interventions [16]. Within the existing literature, this study offers the most general comparison of scaling activity. At the same time, we recognize that we address one aspect of scale, trans-national activity, and focus on identifying the characteristics of programs that achieve TNS status, without being able to reach conclusions about how or why they were able to reach multi-country status.

The characteristics of programs in the study that have achieved trans-national scale differ from single country programs for reasons that may reflect relevant drivers for, and constraints to, geographic replication. TNS programs most commonly emphasize targeted health needs rather than more comprehensive care, provide healthcare delivery support rather than direct clinical care, are private non-profits, and rely particularly heavily on donor funding. Single country programs are more likely than TNS programs to provide comprehensive primary and secondary clinical care, and while they also commonly rely on donor funding, they are also more often able to draw on public financing and/or membership fees.

We draw on the broader literature to consider several factors that may underlie these patterns, including capital and skills intensity, as well as funder preferences. Prior studies suggest that capital-intensive interventions and those that require complex human resource interventions are difficult to scale [9], [16], [17]. Many of the reported scaled programs in our study conduct activities related to marketing and consumer education that require relatively low financial investment and limited human resource needs to achieve increases in output. General primary care, in contrast, is not easily standardized, and thus, is more difficult to scale due to the complexity.

Nonetheless, despite the common difficulties in scaling complex activities, this study found that some programs that provide sophisticated clinical services are able to achieve trans-national scale. One example is OpAsha, which provides TB treatment in India and Cambodia. OpAsha focuses on a single disease area using highly repetitive processes. The broader literature supports the idea that service standardization advances scalability [9], [18], [19], [20].

Preferences of funders, including donors and governments, as well as for-profit status, also, undoubtedly, shape the patterns. Several studies suggest that achieving scale, including trans-national scale, requires financial sustainability [9], [18]. Donors are by far the primary source of funding for the TNS programs in our study. Typically, donors emphasize non-profit, rather than for-profit, or public ventures. In turn, donors commonly have strong preferences for their support. Between 2001 and 2007, one third of all donor funding was targeted for HIV/AIDS, malaria, and tuberculosis [6], which reflect easily measurable Millennium Development Goals [21]. By inference, this implication helps explain why HIV, TB, and malaria, as well as other targeted needs such as, family planning and maternal and child health are the most reported health foci among programs scaled internationally.

Thus, the implications of the results suggest that donor funding can help programs surmount capital barriers so that they can operate in multiple countries, either from the outset or via expansion. The expansion can leverage insights and lessons from one country to help support healthcare activities in multiple settings.

At the same time, donor funding commonly has substantial limits, which can constrain the ability to invest in more general care and capital-intensive activities. The emphasis on targeted health needs, and lesser involvement in general primary care, reflects a limit in the scope of impact of many of the trans-national scaled programs. Changes in disease conditions, and more general health needs, are demonstrating an increased health burden stemming from non-communicable disease and more years lived with disability [22], advancing the need for robust national health systems with broad scope of primary care and universal coverage. At this point, though, it appears that programs that achieve trans-national scale are often vertical approaches that most commonly target particular healthcare needs and/or support rather than carry out clinical activities.

Clearly, these targeted efforts have high potential value in filling critical gaps in specific health services. Nonetheless, it is possible that the vertical approaches may contribute to fragmentation of health services among national health systems [23], [24], with potentially adverse impacts on quality, cost, and outcomes [25]. There is also a risk that pressures to scale up health programs may lead to trade-offs and compromise pro-poor targeting, equity [12], [17], and/or quality [26], which may be particularly problematic in vertical programs. These issues deserve additional research.

The limited involvement by government health agencies in TNS programs undoubtedly reflects their local priorities. Public agencies, which are often most central to providing primary care [27], [28], have mandates to improve health services within their own countries. Nonetheless, the examples of public involvement in trans-national scale, such as the WADRAN, Tanzania-Ghana Health partnerships and the Strengthening Laboratory Management Accreditation program of the CDC (noted earlier) demonstrate paths that are consistent with country-specific mandate, and these examples, too, require further study.

In parallel, we found only limited involvement of for-profit entities in TNS programs, which may reflect the difficulty of achieving profitable operations from complex organizations, particularly when targeted at relatively poor populations. Nonetheless, as we noted earlier, some for-profit ventures have expanded into niches in multiple countries, also meriting further attention.

Despite the constraints, the study suggests that, in addition to the more common targeted support, there are potential paths to achieving trans-national scale of general care. The TNS programs providing general primary care tended to be founded more recently (median of 2010/2011 v. 2006 in the full set of programs), often as donor-supported non-profits, possibly reflecting a growing willingness to invest in broader care. The general care programs existed across all three continents, with a slightly higher median number of countries than the overall TNS population (4 v. 3). Underlying this ability to achieve greater TNS breadth, programs providing and supporting general care often involved telemedicine and other telecom-supported services. Examples include the Heberden Telemedicine System that connects providers in Africa and Haiti with physicians in the U.S., Israel, and Europe, and the Africa Teledermatology Project present in six countries in Sub-Saharan Africa. Such programs reflect increasing ability to apply information technology to healthcare services.

### Strengths and limitations

There are both strengths and limits to this study. The research is based on a large dataset with substantial information on nearly 1,200 programs, of which, 116 have achieved trans-national scale. This allows for statistical power to make comparisons to single-country programs, covering focal health areas, program activities, funding sources, and legal status, as well as country locations, urban-rural coverage, and founding years.

At the same time, several limits point to the need for future research. Potential selection biases may affect inclusion in the database. The study does not examine replication of innovations across organizations. The analysis cannot distinguish between a presence in a country and high-impact operations in that country. In turn, it was not possible to examine the quality of TNS and single country programs to understand whether trade-offs were being made between scale and quality, or to determine whether programs were replicated equally in all countries. We cannot determine the number of patients treated or population coverage. The cross-sectional design provides a snapshot of programs at one point in time, without providing trend information. Further research is needed to understand the goals of the programs, as well as structures and processes that must be in place to support successful multi-country replication, the major determinants of successes and failures in scaling up, and the trade-offs and strategic choices involved in achieving TNS status. Such research will need to examine the influence of politics and socio-cultural norms on the scale process as well as the trade-offs in program mandates and pro-poor targeting that may be necessitated as programs attempt to scale.

## Conclusions

Understanding TNS is important conceptually, empirically, and in practice. Conceptually, TNS is a meaningful indicator of how broadly a program is able to spread its reach. For empirical healthcare measurement, TNS is an objective measure of scale that is comparable across hundreds of organizations. For healthcare practice, TNS is a relevant measure of replicability, demonstrating which program models are conducive to being transplanted in different contexts.

Program managers and donors can benefit from knowing the characteristics of healthcare programs that achieve trans-national scale. The study suggests that certain processes can help advance TNS such as provider training, logistics support, and supply chain enhancements. The study also offers insights on the kinds of healthcare activities that are more amenable to scale up.

At its core, the study offers two contrasting implications for health services policy and practice when targeting the poor in LMICs. Firstly, most TNS programs in the study deliver disease-specific vertical interventions rather than more comprehensive clinical care. Secondly, the data reveals that some TNS programs have been able to scale clinical care, demonstrating that while scaling clinical care is challenging, it is possible. Examining how clinical care can be scaled up warrants further examination as it is an integral component of health services delivery. Most generally, this study is part of global efforts to understand how scale is achieved in practice, with the goal of helping health services scale effectively to improve population health.

## References

[1] Jamison DT, Breman JG, Measham AR, Alleyme G, Claeson M, et al. (2006) Disease Control Priorities in Developing Countries Project, 2^nd^ Edition. Oxford University Press and the World Bank. 21250309

[2] Alliance for Health Policy and Systems Research (2004) Strengthening health systems: The role and promise of policy and systems research. Available: http://www.who.int/alliance-hpsr/resources/Strengthening_complet.pdf. Accessed 2013 Feb 9.

[3] Saksena P, Xu K, Elovainio R, Perrot J (2012) Utilization and expenditure at public and private facilities in 39 low-income countries. Trop Med Int Health 17(1): 23–35 10.1111/j.1365-3156.2011.02894 22008480

[4] Karamchandani A, Kubzansky M, Frandano P (2009) Emerging markets, emerging models: Market-based solutions to the challenges of global poverty, Monitor Group. Available: http://web.mit.edu/idi/idi/India%20Emerging%20markets%20Emerging%20models_MIM.pdf. Accessed 2013 Mar 16.

[5] Bhattacharyya O, Khor S, McGahan A, Dunne D, Daar A, et al (2010) Innovative health service delivery models in low and middle income countries – what can we learn from the private sector? Health Research Policy and System 8(1): 2 10.1186/1478-4505-8-24 PMC3236300 20630108

[6] Bloom G, Ainsworth P (2010) Beyond scaling up: Pathways to universal access to health services. STEPS Centre. Available: http://opendocs.ids.ac.uk/opendocs/bitstream/handle/123456789/2278/Beyond%20Scaling%20Up.pdf?sequence=1. Accessed 2013 Mar 20.

[7] Credit Suisse (2012) Investing for impact. Credit Suisse. Available: https://infocus.creditsuisse.com/data/_product_documents/_shop/33096/investing_for_impact.pdf. Accessed 2013 Apr 22.

[8] Subramanian S, Naimoli J, MatsubayashiT, Peter DH (2011) Do we have the right models for scaling up health services to achieve the Millennium Development Goals? BMC Health Services research 2011 11: 336. 10.1186/1472-6963-11-336 PMC3260120 22168915

[9] Cooley L, Khol R (2006) Scaling up: From vision to large-scale change a management framework for practitioners. Management Systems International. Available: http://www.msiworldwide.com/files/scalingup-framework.pdf. Accessed 2012 Dec 8.

[10] Simmons R, Fajans P, Ghiron L, eds. (2009) Scaling Up Health Services Delivery: From Pilot Innovations to Policies and Programmes. Worlds Health Organization, Geneva. Available: http://whqlibdoc.who.int/publications/2007/9789241563512_eng.pdf. Accessed 2012 Nov 13.

[11] Centre for Health Market Innovations (2008) Sproxil. Available: http://healthmarketinnovations.org/program/sproxil. Accessed 2013 Apr 27.

[12] Waddington C (2012) Scaling up health services: Challenges and choices. Available: http://www.hlsp.org/LinkClick.aspx?fileticket=kZ4sevUUp0g%3D&tabib=1570. Accessed 2013 Mar 11.

[13] Kamal-Yanni MM, Potet J, Saunders PM (2012) Scaling-up malaria treatment: A review of the performance of different providers. Malaria Journal 11: 1–10 10.1186/1475-2875-11-414 23231707 PMC3547718

[14] Lunk C, Boyce G, Flisher AJ, Kafaar Z, Dawes A (2009) Scaling up child and adolescent mental health services in South Africa: Human resource requirements and cos. Journal of Child Psychology and Psychiatry 50: 1121–1130 10.1111/j.1469-7610.2009.02078 19243477

[15] Knippenberg R, Lawn JE, Darmstadt GL, Begkoyian G, Fogstad H, et al (2005) Systematic scaling up of neonatal care in countries. The Lancet 635: 1087–1098. 10.1016/S0140-6736(05)71145-4 15781104

[16] Johns B, Torres TT (2005) Costs of scaling up health innovations: A systematic review. Health Policy and Planning 20(1): 1–13. 15689425 10.1093/heapol/czi001

[17] Mangham LJ, Hanson K (2010) Scaling up international health: What are the key issues? Health Policy and Planning 25: 85–96 10.1093/heapol/czp066 20071454

[18] Yamey G (2011) Scaling up global health interventions: A proposed framework for success. PLOS Medicine 8(6): 1–5. 10.1371/journal.pmed.1001049 PMC3125181 21738450

[19] World Bank (2003) Scaling –UP the Impact of Good Practices in Rural Development. A working paper to support implementation of the World Bank's Rural Development Strategy. Available: http://www.wds.worldbank.org/servlet/WDSContentServer/IW3P/IB/2004/01/30/000160016_20040130163125/Rendered/PDF/260310White0co1e1up1final1formatted.pdf. Accessed 2013 Feb 3.

[20] Tung E, Bennett S (2014) Private sector, for-profit health providers in low and middle income countries: can they reach the poor at scale? Globalization and Health 10(52).. 10.1186/1744-8603-10-52 PMC4094686 24961496

[21] United Nations (2002) Millennium Development Goals. Available: http://www.un.org/millenniumgoals/. Accessed 2013 May 10.

[22] Murray CJL, Vos T, Lozano R, Naghavi M, Flaxman AD, et al (2012) Disability-adjusted life years (DALYs) for 291 diseases and injuries in 21 regions, 1990-2010: A systematic analysis for global burden of disease study 2010. Lancet 380(9859): 2197–2223 10.1016/S0140-6736(12)61689-4 23245608

[23] McIntyre D, Garshong B, Mtei G, Meheus F, Thiede M, et al (2008) Beyond fragmentation and towards universal coverage: Insights from Ghana, South Africa and the United Republic of Tanzania. Bull World Health Organ 86(11): 817 Available: http://www.who.int/bulletin/volumes/86/11/08-053413/en/ Accessed 2013 Feb 19.. 10.2471/BLT.08.053413 PMC2649570 19030693

[24] Yu D, Souteyrand Y, Banda MA, Kaufman J, Perriëns JH (2008) Investment in HIV/AIDS programs: Does it help strengthen health systems in developing countries? Globalization and Health 4: 1–10. 18796148 10.1186/1744-8603-4-8 PMC2556650

[25] Enthoven AC (2009) Integrated care delivery systems: the cure for fragmentation. American Journal of Medical Care15(10): : 284–90 Available: http://www.ncbi.nlm.nih.gov/pubmed/20088632. Accessed 2013 Feb 22.

[26] Government of Tanzania and Clinton Foundation (2008) Tanzania Pilot ACT Subsidy: Report on Findings New York: Clinton Foundation. Available: http://www.clintonhealthacces s.org/news-and-information/Tanzania-pilot-act-subsidy-report. Accessed 2012 Nov 22.

[27] World Health Organization (2008) The World Health Report 2008: Primary Health Care: Now More Than Ever. World Health Organization.

[28] World Health Organization (2005) Sustainable health financing, universal coverage and social health insurance 2005. World Health Assembly Resolution WHA 58.33.Geneva: WHO. Available: http:www.who.int/providingforhealth/topics/WHA58_33-en.pdf. Accessed 2013 Feb 19.

